# Effect of Recurrent Selection on Drought Tolerance and Related Morpho-Physiological Traits in Bread Wheat

**DOI:** 10.1371/journal.pone.0156869

**Published:** 2016-06-14

**Authors:** Ramya P, Gyanendra Pratap Singh, Neelu Jain, Pradeep Kumar Singh, Manoj Kumar Pandey, Kavita Sharma, Arun Kumar, Harikrishna, Kumble Vinod Prabhu

**Affiliations:** Division of Genetics, ICAR-Indian Agricultural Research Institute, New Delhi, Delhi, India; National Institute of Plant Genome Research, INDIA

## Abstract

Drought is one of the major abiotic stresses affecting wheat yield. A recurrent selection program was conducted to improve the drought tolerance and yield of bread wheat using drought tolerant advanced breeding lines from a drought tolerant x susceptible cross (HI 1500 x HUW 510). The parental lines were evaluated for yield, biomass and harvest index (HI) in addition to the drought adaptive traits like Canopy Temperature (CT), chlorophyll content and Normalized Difference Vegetative Index (NDVI). After three rounds of recurrent selection, the half sib progenies exhibited a marked reduction in CT, chlorophyll content and biomass; whereas improvement was noted for yield, HI and NDVI. Drought tolerance of the half-sib population appeared enhanced as indicated by drought indices and grain yield. Compared to base population, half-sibs showed better HI, grain filling and a significant (17.1%) increase in grain yield under water stress conditions. Cooler canopies and increased early vigour might have contributed to drought tolerance. A favourable combination of gibberellin sensitive and insensitive *Rht*alleles was observed in the recombinant progenies. However, increased yield under water stress had a negative trade off in reduction of biomass. The study also identified potential lines with high yield and drought tolerance for subsequent varietal development for water limited areas.

## Introduction

Climate change and shrinking water resources have catapulted drought as the most important abiotic stress affecting the productivity of field crops world-wide [1]. Wheat (*Triticumaestivum*) is an important food crop in India often seriously affected by high temperatures and water stress. In India, even though 90% of the wheat is grown under irrigated conditions, only one-third receives full irrigation while the rest is cultivated under partial irrigation [2]. It is likely that water will become a limiting factor for sustained production of wheat in India [3]. Wheat yields have been projected to reduce by an amount of -2292.6 (1000 metric ton) under irrigated conditions due to the impact of climate change and associated water scarcity by the year 2030 [4]. Exposure to temperatures higher than 34°C leads to accelerated senescence which results in poor grain filling and hence low grain yields. Thus with every 2°C rise in temperature the associated wheat yield loss is estimated as much as 50% for some sowing dates [5]. The genes and metabolic pathways that confer drought tolerance in crop plants can be unravelled by modern genomics and genetic approaches coupled with advanced techniques for precision phenotyping and molecular breeding methods [6]. The recent advances in plant physiology lead to the development of precise phenotyping tools and techniques which help in drought tolerance breeding. Due to the large genotype x environment interactions and low heritability, selection for improved performance under drought conditions based on grain yield alone is considered to be ineffective [7]. In such situations it is advocated that secondary traits with increased genetic variance under stress conditions can be incorporated to increase selection efficiency provided such traits have high heritability, greater adaptive value and relatively easy to measure [8,9]. The data from Semi Arid Wheat Yield Trials (SAWYT) in South Asia over the years using lines bred at CIMMYT, Mexico using conventional and physiological trait (PT) approaches has shown that PT lines were superior yielding with higher grain yield and cooler canopy temperatures (CT) [10].

Breeding for drought and terminal heat tolerance is an important abiotic stress aspect of Indian wheat improvement program. The breeding strategy developed by CIMMYT for drought and heat tolerance is rooted on the interplay of breeding, molecular biology and physiology with due emphasis on fine dissection of morpho-physiological traits through precision phenotyping [11]. The relationship between grain yield under drought stress conditions and Canopy Temperature (CT) is well established [12–14]. Cooler canopy temperature (CT) is associated with both drought and heat tolerance [15]. ‘Stay-green’ is a drought adaptive trait characterised by distinct green leaf phenotype during grain filling under terminal drought [16]. Since drought induces early senescence reports indicate that flag leaf sheaths serve a vital role in grain filling by storing and transporting assimilates to the developing grains of wheat plants [17]. Flag leaf chlorophyll content measured with portable instrument (SPAD meter) is considered as an indication of ‘stay-green’ or delayed senescence [18]. ‘Stay-green’ in the post anthesis phase is reported to be associated with drought tolerance in several crops [19–20] and is utilized for breeding drought tolerant cultivars in wheat [21]. CT, chlorophyll content and NDVI (Normalized Difference Vegetative Index) have been effectively combined for rapid screening of drought and heat tolerance in wheat [22] and several PT lines [10] thus developed are being utilized in Indian wheat breeding program.

Recurrent selection is a population improvement strategy which utilizes multiple parents in the crossing program to accumulate favourable alleles while maintaining genetic diversity. Although widely studied in maize [7, 23], the strategy has also been applied to other crops like barley [24, 25]; rice [26]; pearl millet [27] and soybean [28]. In wheat, recurrent selection has been successfully applied to improve percentage of grain protein [29–30] kernel weight [31–32] and grain yield [33–34]. Recurrent selection for drought tolerance has been reported in maize [23, 35–37]. In wheat, recurrent selection was practiced to breed lines with greater early vigour so as to have increased water and nutrient efficiency [38]. This paper reports the implementation of recurrent selection strategy based on physiological and morphological traits related to drought tolerance for identification of superior drought tolerant progenies with high yield as well as the assessment of genetic gain and response to selection following three cycles of recurrent selection.

## Materials and Methods

### Experimental conditions

The experiment was conducted during 2007–2015 in India at Delhi whereas multi location trials for selection of parents were conducted at Delhi, Ludhiana, Powarkheda and Pune in the rabi season of 2011–2012. For recurrent selection, crossing of selected lines was done at Delhi, while the crossed seeds were raised at LahaulSpiti (Directorate of Wheat Research Station, DalangMaidan). Details of experimental locations are given as a supplementary file (S1 Table). The IARI experimental farm where bulk of the work was done comes under north western plain wheat belt having semi-arid sub tropical climate with clay loam textured alluvial soil, mildly alkaline pH and low organic carbon content. The experiment was laid out in alpha-lattice design with two treatments and two replications [39]. The treatments given were restricted irrigation (single life saving irrigation during crown root initiation (CRI)) and rainfed (with no irrigation). The initial moisture content of both irrigated and rainfed fields in Delhi at the time of sowing was 30%, however the average moisture content of irrigated field was 25% whereas that of rainfed field was 12% at a depth of 60cm. The lines were planted manually with gross plot size of 0.46 x 2.5m with rows at 23 cm apart (3 rows). The standard management practices for wheat were followed. The drought tolerant commercial varieties HD2987, HD3043 and the original parents (HI1500 and HUW510) were used as checks.

### Base population

The original population consisting of 157 lines was derived from the cross HI 1500 x HUW 510. HI 1500 is a known source of drought tolerance derived from the cross “HW 2002*2/Strempallli/ PNC 5”; while HUW 510 is a drought susceptible variety developed from cross HD 2278/HUW 234//DL 230–16. The parents were thoroughly screened for drought tolerance [40] using physiological and molecular tools. The lines developed were carried over from F_2_ to F_5_ by modified bulk pedigree method without selection both under restricted irrigation (RI) and rainfed (RF) conditions.

### Selection of parents for recurrent selection

The F_4_lines, parents (HI 1500 and HUW 510) and check variety (HD 2987) were evaluated at Delhi, Ludhiana, Powarkheda and Pune. The experiment was laid out in alpha-lattice design with the same plot size, treatment and replications during *rabi*season of 2011–2012. Based on drought tolerance, stability across locations, physiological characterisation and grain yield, twelve F_4_ plants were selected as parents for the recurrent selection program [S2 Table and S1 Fig].

### Recurrent selection strategy

#### Cycle zero

The total (base) F_5_ population, parents and checks (HD 2987 and HD 3043) were planted in the field in alpha lattice design with the same plot size, treatments and replication at Delhi. The twelve selected F_5_ plants were intermated among themselves roughly in half-sib fashion. (Details of crossing pattern given as S3 Table.) There were 11 half-sib families initially of which one poor performing family (hs11) with very low F_1_ seed set of single cross (11x117) was removed to arrive at the final 10 half-sib families. The number of crossed lines in each family varied from 4 to 11. The total number of crossed lines involved was 75; in addition all the parental lines were selfed. For rapid generation advancement the crossed seeds were grown at Lahaul-Spiti and subjected to phenotyping using physiological and yield traits.

#### Cycle one to three

The 75 crossed lines from Lahul-Spiti were planted in the field along with the base population (157 lines carried forward as bulks) and check in the same experimental design with same plot size, treatment and replications. The lines were characterised again for physiological and yield traits. The best plants based on phenotyping data from each line in each family were intermated again based on previous crossing pattern and seeds raised at Lahaul-Spiti. This exercise was repeated again for second and third season to generate the final cycle of recurrent selection.

### Physiological traits

Data collection was done on the basis of 10 plants per plot for crossed lines, checks (HD 2987 and HD 3043) and parental lines (HI 1500 and HUW 510). The physiological traits measured were Canopy Temperature (CT) at vegetative and reproductive stages, chlorophyll content at reproductive stage and NDVI at weekly intervals during plant growth. Canopy temperature was measured with the help of hand held infrared thermometer (Kane May Model Infratrace 8000, USA). Leaf chlorophyll content was measured with the help of handheld chlorophyll meter (SPAD 502, Konica Minolta, Osaka, Japan) as an indicator of ‘stay-green’ trait. Data was taken on 10 flag leaves and averaged per plot. NDVI was measured with the help of hand held Trimble GreenSeeker.

#### Harvest Index (HI), biomass and grain yield

Biomass was calculated as above ground plant parts yield while HI is calculated as the ratio of grain yield to the total aboveground biomass [41]. In addition grain yield per plot was also taken.

#### Data analysis

The analysis of variance for each cross was done separately using linear mixed models keeping all the other effects except genotype and treatment (stress) as random, with the help of software package GENSTAT version 16 (VSN International Ltd, UK). Broad sense heritability, genetic advance, genetic gain %, gain over F_5_ parents and best check (HD 3043) were calculated in Excel work sheet. Standard T- tests were devised to test the significance of difference between the means of half-sib families against the base population and the check (HD 3043). For comparing the performance of individual half-sib families against base population and check varieties (HD2987 and HD3043) Dunnett’s tests were done.

Broad sense heritability was derived from 3 way model (genotype x treatment x year) by modifying the equation [42].

HBS={σ2G[σ2G+(σ2GTt)+(σ2GYy)+(σ2GTYty)+(σ2eryt)]}×100

where σ^2^ G = genotypic variance, σ^2^ GT = variance due to genotype x treatment interaction, σ^2^ GY = variance due to genotype x year interaction, σ^2^ GTY = variance due to genotype x treatment x year interaction and σ^2^ e = error variance. The terms r, y and t denotes replication, year and treatment respectively.

The genetic advance (GA) was calculated [43] from the following formula: *GA* = *K* × *σP* × *H*_*BS*_; while Genetic Gain % was calculated as (GA/Mean) 100; where K is the selection differential taken as 2.06 at 5% level of selection; σ P standard deviation of the phenotypic variance and H_BS_ is the heritability in broad sense.

Correlation analysis was done using the adjusted means obtained from analysis of variance for each cross. Yield BLUPs were calculated using lme4 package [44] in R software using REML criterion of convergence fitting the formula

Yield~(1 | Progeny) + (1 | Treatment) + (1 | Year) + (1 | Rep %in% Treatment: Year) + (1 | Progeny: Treatment) + (1 | Progeny: Year)

Different drought indices were worked out [45–48] for ranking the crosses according to drought tolerance using the mean yields under RI and RF (Y _(ns)_ and Y_(s)_) as well as the yield of individual crosses under RI and RF (Yi _(ns)_ andYi_(s)_). The indices used are;

1. Stress tolerance (TOL) = Yi_(ns)_ − Yi_(s_).
2. Mean Productivity Index (MPI) = Yi_(ns)_ + Yi_(s)_/2
3. Mean Relative Performance $\left(\text{MRP}\right)=\;\left(\frac{{\text{Yi}}_{\left(\text{s}\right)}}{{\text{Y}}_{\left(\text{S}\right)}}\right)+\;\left(\frac{{\text{Yi}}_{\left(\text{ns}\right)}}{{\text{Y}}_{\left(\text{ns}\right)}}\right)$
4. Relative Efficiency Index (REI) = (Yi(s)/Y(S)) × (Yi(ns)/Y(ns))
5. Geometric Mean Productivity (GMP) = √ Yi(s) × Yi(ns)
6. Stress Tolerance Index (*STI*) = (*Yi*(*ns*) ×*Yi*(*s*))/*Y*(*ns*)2)

## Results

### Selection of F_5_ lines as parents

**Fig 1:**
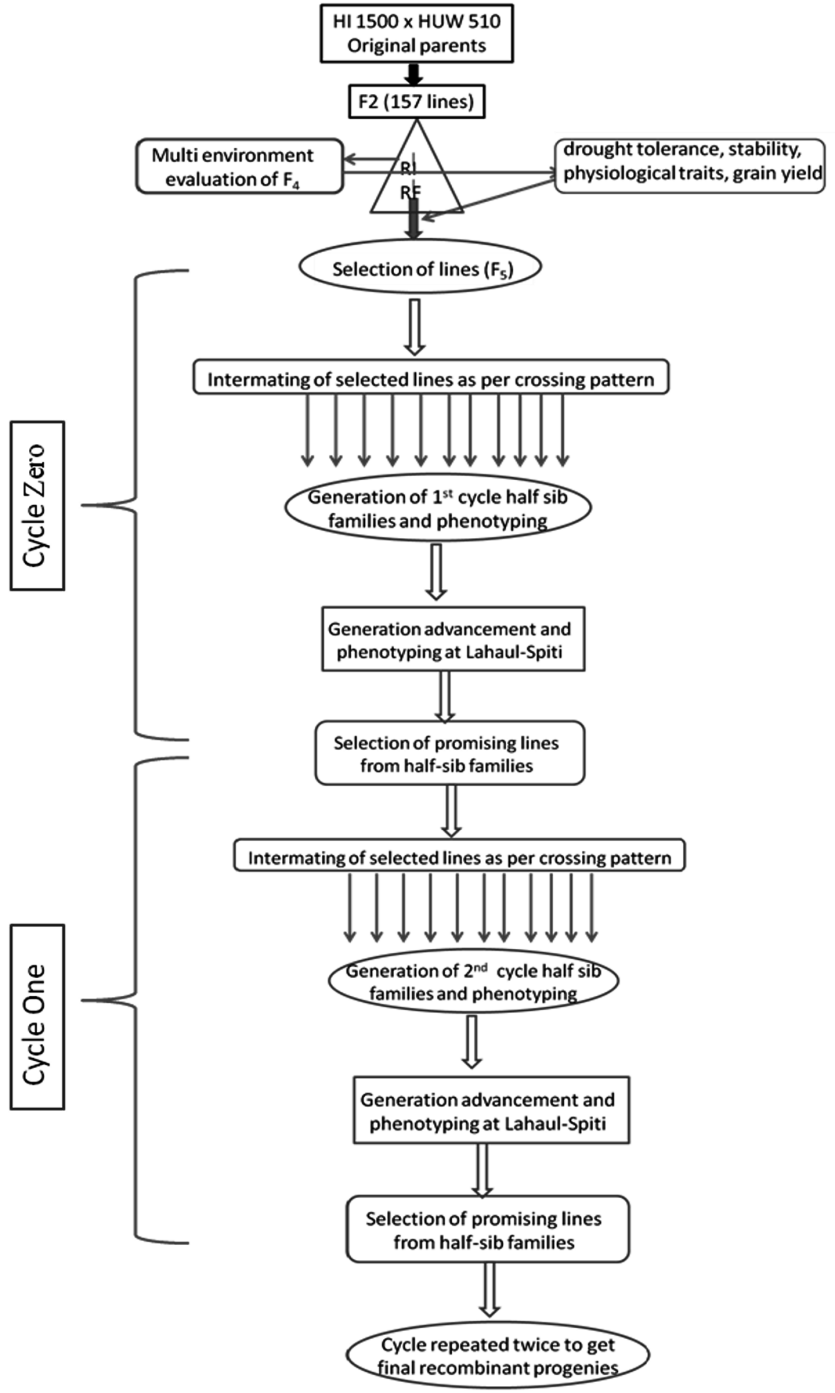
Scheme of recurrent selection. At the end of each cycle the progenies with higher yield and physiological attributes were selected as the base material for next cycle.

The F_5_ lines were selected based on yield, drought tolerance, stability across locations and with a fine balance of related physiological traits. Biplots were used to assess the stability whereas drought tolerance was worked out using indices like TOL, GMP, MPI, STI, REI and MRP. Since the index GMP was found to have significant positive correlation with all the other indices it was used as the yardstick of drought tolerance. The best 50 lines were identified on the basis of stability and drought tolerance from which 12 lines were further selected as parents for recurrent selection (scheme given in Fig 1) based on grain yield and physiological aspects i.e. with high chlorophyll content, biomass, HI and low canopy temperatures. Details are given as supplementary files [S2 Table, S1 Fig].

### Effect of recurrent selection on the population

There were significant differences among the half-sib families with respect to maturity and anthesis. Days to natural maturity ranged from145-155 while anthesis happened between 82–106 days after planting. Significant changes were recorded for all the traits studied as a result of recurrent selection.

### Biomass

After three rounds of recurrent selection there was considerable reduction in the biomass. The mean value for biomass in the F_5_ population was 2134 kg whereas in the half sib population it was reduced to 1634 kg. The broad sense heritability for biomass in the base population was 37.86% while that of half sib population is 23.12%. The mean genetic gain % after recurrent selection was 18.35. Among the half sib families; the range of biomass observed was 1413 to 1719 kg; the highest and lowest values noticed in half sib families 35 (hs35) and 116 (hs116) respectively. The family hs35 also recorded the highest genetic advance and genetic gain. The heritability also ranged between 11.7% (hs68) to 43.01% (hs65). When the original F_5_ parental values are considered the reduction in biomass after recurrent selection ranged from 0.23% to 43.77% while the reduction over best check was 9.65 to 25.7%. The significance of both was confirmed by t test. The details of genetic gain and heritability for each half sib family are given in Table 1.

**Table 1:** Mean, percentage genetic gain over mean, percentage gain over F5 and percentage gain over check HD3043 for biomass and chlorophyll content. T (p) and T(c) are the T tests conducted to check the significance of difference between half sib families with base population and check HD3043 respectively for biomass and chlorophyll content.

	BIOMASS				CHLOROPHYLL CONTENT			
Line	Mean	Genetic Gain%	Gain over F5%	Gain over check %	Mean	Genetic Gain%	Gain over F_5_%	Gain over check
base	2134	22.59			50.27	8.97		
hsib	1634	18.35			48.55	4.47		
hs26	1565	13.65	-32.89	-17.74	49.40	5.42	6	8.6
hs35	1719	49.82	0.233	-9.65	47.19	3.52	-1.44	3.7
hs39	1602	9.38	-11.29	-15.80	48.95	3.05	-1.73	7.6
hs48	1582	7.65	6.25	-16.85	49.25	5.23	4.76	8.2
hs65	1647	9.94	-5.72	-13.43	48.73	2.86	2.24	7.1
hs68	1716	3.45	3.81	-9.80	48.48	4.02	-4.75	6.5
hs79	1605	7.97	-35.02	-15.64	48.29	11.27	-2.4	6.1
hs86	1714	5.34	-10.96	-9.91	48.40	2.07	-1.4	6.4
hs91	1678	6.7	-4.17	-11.80	48.25	3.83	2.2	6.0
hs116	1413	3.6	-43.77	-25.73	48.51	2.41	-4.1	6.6
T test	T(p) = 0.012*	T(c) = 0.002**			T(p) = 0.68	T(c) = 0.002**		

*and** indicate the significance level.

### Canopy Temperature

After recurrent selection there was marked decrease in canopy temperature of half sib population at both reproductive and vegetative stage. There was an average reduction of 4.47°C at vegetative stage and 1.28°C at reproductive stage compared to the F_5_ base population. Among the half sib families the lowest CT at vegetative stage was recorded for hs39 and hs68 respectively (14.15°C). The heritability CT at vegetative stage (CT-Veg) in the base population was 36.58 while that in the half sib population was 25.04. For CT-Veg maximum genetic gain was also shown by family hs65 followed by hs39 and hs68 while maximum gain over F_5_ parent was shown by family hs26 followed by hs68. Maximum gain over check was also shown by hs 68 and both the t tests were highly significant, details given in Table 2.

**Table 2:** Mean, percentage genetic gain over mean, percentage gain over F_5_ and percentage gain over check HD3043 for Canopy Temperature (CT) at vegetative stage and reproductive stage. T (p) and T(c) are the T tests conducted to check the significance of difference between half sib families with base population and check HD3043 respectively for CT.

CT (Vegetative)					CT (Reproductive)			
Line	Mean	Genetic Gain%	Gain over F_5_%	Gain over check	Mean	Genetic Gain%	Gain over F_5_%	Gain over check%
base	18.78	3.83			27.23	2.64		
hsib	14.31	2.87			25.95	1.9		
hs26	14.33	3.28	-25.71	-9.48	26.01	1.9	-2.11	-5.52
hs35	14.50	1.82	-22.5	-8.40	25.93	1.9	-5.19	-5.81
hs39	14.15	3.73	-6.78	-10.61	25.81	4.82	-2.35	-6.25
hs48	14.22	1.52	0.8	-10.17	25.96	1.15	1.05	-5.70
hs65	14.25	4.26	-1.04	-9.98	25.89	2.8	-3.54	-5.96
hs68	14.15	3.56	-23.92	-10.61	25.90	0.58	-6.13	-5.92
hs79	14.38	3.6	-21.55	-9.16	25.91	2.24	-4.71	-5.88
hs86	14.44	0.63	-4.94	-8.78	26.09	1.54	-2.61	-5.23
hs91	14.21	2.7	-1.04	-10.23	25.96	2.16	0.426	-5.70
hs116	14.41	1.4	-23.15	-8.97	25.96	2.7	-5.6	-5.70
T test	T(p) = 0.001**	T(c) < .00001***			T(p) = 0.001**	T(c)<0.00001***		

** and *** indicate the significance level.

For CT at reproductive stage, the broad sense heritability recorded for half sib family (37.7%) was higher compared to the base population (36.58%). Maximum H_BS_ obtained was 55.7% in family hs116. Maximum genetic gain was recorded in the family hs39 followed by hs116, while the lowest genetic gain was for the family hs68. All the families reported significant gains over check variety also (Table 2).

### Chlorophyll content

The mean chlorophyll content after selection was found to be reduced by 3.42% after selection (Table 1). All of the half sib families were having chlorophyll content less than that of mean value of base population. Compared to the original F_5_values four families (hs26, hs48, hs65 & hs91) gained positively. The heritability of the base population was 47.95% and that of half sib population was 36.15%. Some half sib families exhibited low heritability with the range being 22.95–68.05%. The highest heritability recorded (68.05%) was for hs79. This family recorded highest genetic gain also when compared to the mean value (11.27%). Positive selection differential was noticed only in four half sib families (hs26, hs39, hs48 and hs65). Significant reduction in chlorophyll content of half sib families was noticed on comparison with check.

### NDVI

There was a positive increase in NDVI values after selection. Six out of ten half sib families were having higher NDVI values than the base population (Table 3) while all the families gained significantly when compared to check HD 3043. Most of the families showed higher heritability than the base population. Percentage genetic gain was maximum in the family hs65 (57.65%) and minimum in hs35 (1.3%). The family hs79 had zero selection differential, as the NDVI value was same as mean value of population. Between the rest nine families, five were having positive value for selection differential while four had negative value. Maximum gain over F5 parental value was noted in hs39 while least gain was observed in families’ hs26 and hs116.

**Table 3:** Mean, percentage genetic gain over mean, percentage gain over F5 and percentage gain over check HD3043 for HI and NDVI. T (p) and T(c) are the T tests conducted to check the significance of difference between half sib families with base population and check HD3043 respectively for HI and NDVI.

	HI				NDVI			
Line	Mean	Genetic Gain%	Gain over F_5_%	Gain over check %	Mean	Genetic Gain%	Gain over F_5_%	Gain over check %
base	0.308	18.4			0.508	2.42		
hsib	0.385	10.26			0.510	18.24		
hs26	0.394	7.7	45.93	5.07	0.507	4.39	-1.17	1.40
hs35	0.336	8.72	5	-10.40	0.501	1.3	1.21	0.20
hs39	0.400	11.54	14.3	6.67	0.517	3.33	7.01	3.40
hs48	0.420	4.62	44.83	12.00	0.513	2.75	2.6	2.60
hs65	0.403	5.9	8.92	7.47	0.513	57.65	-0.97	2.60
hs68	0.380	9.23	31.03	1.33	0.516	1.96	1.98	3.20
hs79	0.394	5.64	23.13	5.07	0.510	2.43	-0.59	2.00
hs86	0.371	1.8	12.42	-1.07	0.512	3.92	1.8	2.40
hs91	0.370	3.85	12.12	-1.33	0.503	4.31	-0.59	0.60
hs116	0.411	8.46	37	9.60	0.507	3.33	-1.17	1.40
T test	T (p)<0.001***		T(c) = 0.31		T(p) = 0.062	T(c) = 0.025*		

* and *** indicate the significance level.

### Harvest Index (HI)

There was a significant positive increase in HI after subjecting the population to recurrent selection (Table 3). Six families were having higher HI value than the mean with the highest value measured being 0.42 in hs48. The broad sense heritability value of half sib population and that of families were lower than that of base population. Highest heritability for the trait was observed in hs79 (34.15%). Maximum genetic gain was shown by family hs39 while least gain was in hs86. However, when compared to theF_5_parental values the gain was maximum in hs26 followed by hs48.

### Yield

**Fig 2:**
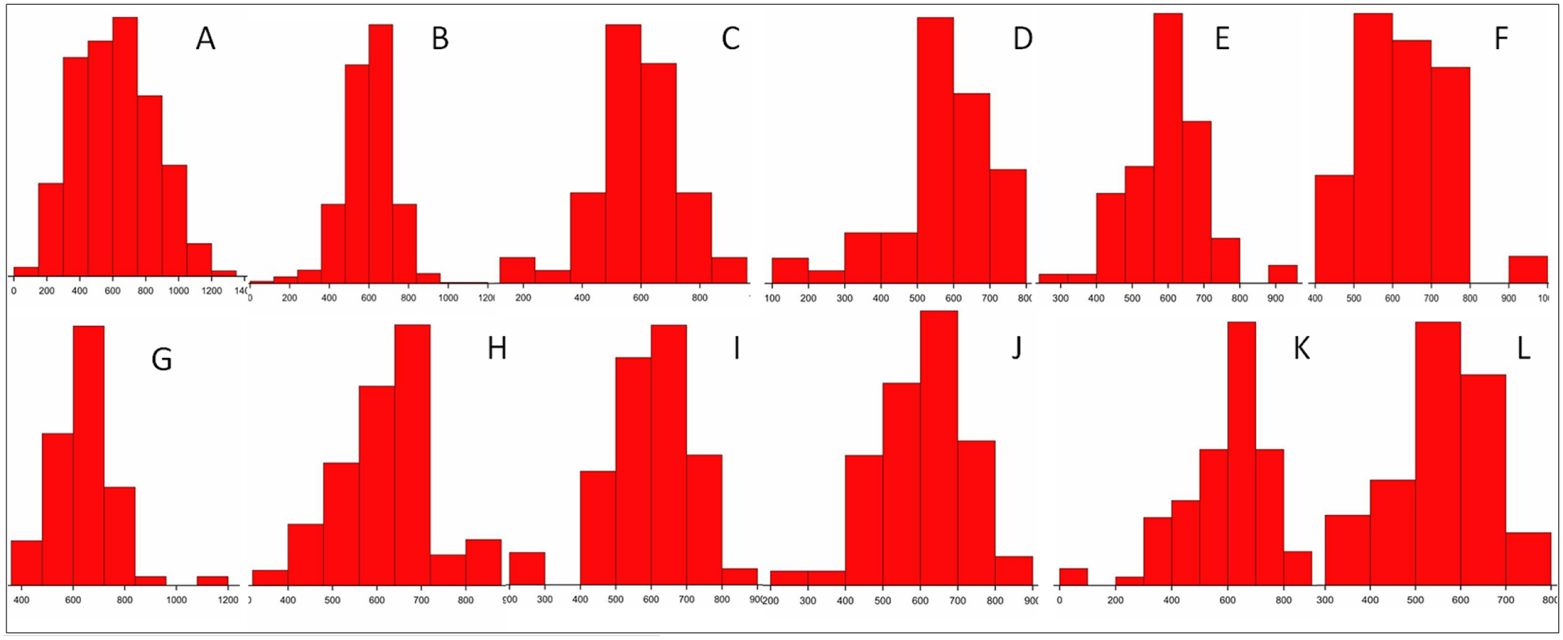
Distribution of yield in base as well as half-sib population. ‘A’ indicates base population; ‘B’ is the half-sib population while C to L are the individual families in the order hs26, hs35, hs48, hs65, hs68, hs79, hs86, hs91 and hs116. The lines in base/ half-sib/individual families are represented in Y-axis while the corresponding grain yield in grams is give in X-axis.

As a result of recurrent selection there was a 17.1% yield increase in the half sib population compared to the base population. Fig 2 represent the distribution of the trait in the base, half sib (all) and individual families. All the families showed very highly significant differences for yield compared to the base population (Table 4). Maximum yield was in family hs65 (640.1g) with a net increase of 24.8% over the base population. This family also recorded maximum gain over the F_5_ parental value. For yield, heritability of half sib population (31.22%) was slightly higher than the base population (29.81%). Maximum value of heritability observed was 38.07 in hs39 while least in hs86 (11.03). The average yield of rest 145 lines carried forward as pedigree bulks at the end of breeding program was 585.5 g whereas that of half-sib population was 601.2 g.

**Table 4:** Range, mean,broad sense heritability(H_(BS)_), genetic advance (GA), percentage genetic gain over mean, percentage gain over F5 and percentage gain over check HD3043 for yield. T (p) and T(c) are the T tests conducted to check the significance of difference between half sib families with base population and check HD3043 respectively for yield.

YIELD							
Line	Range	Mean(g/plot)	H_(BS)_	GA	GeneticGain%	Gain over F_5_%	Gain over check %)
base	1166	513.4	29.81	74.84	14.58		
hsib	1194	601.2	31.22	57.4	9.55		
hs26	810	594.8	31.15	68.08	11.32	20.16	-5.92
hs35	580	566.0	37.11	80.49	13.4	7.4	-11.31
hs39	675	600.9	38.07	58.57	9.74	17.11	-4.84
hs48	522	639.2	36.45	72.74	12.1	11.98	1.44
hs65	821	640.1	33.96	56.47	9.4	23.1	1.58
hs68	485	617.5	27.9	40.98	6.82	10.47	-2.02
hs79	587	599.0	19.5	29.57	4.92	17.3	-5.18
hs86	572	609.2	11.03	30.91	5.14	6.5	-3.41
hs91	888.9	619.0	24.24	48.75	8.11	11.93	-1.78
hs116	475	563.5	29.79	46.17	7.68	2.45	-11.80
T TEST	T(p)<0.0001	T(c) = 0.112					

### Superiority of new lines for drought tolerance, yield and related traits

**Fig 3:**
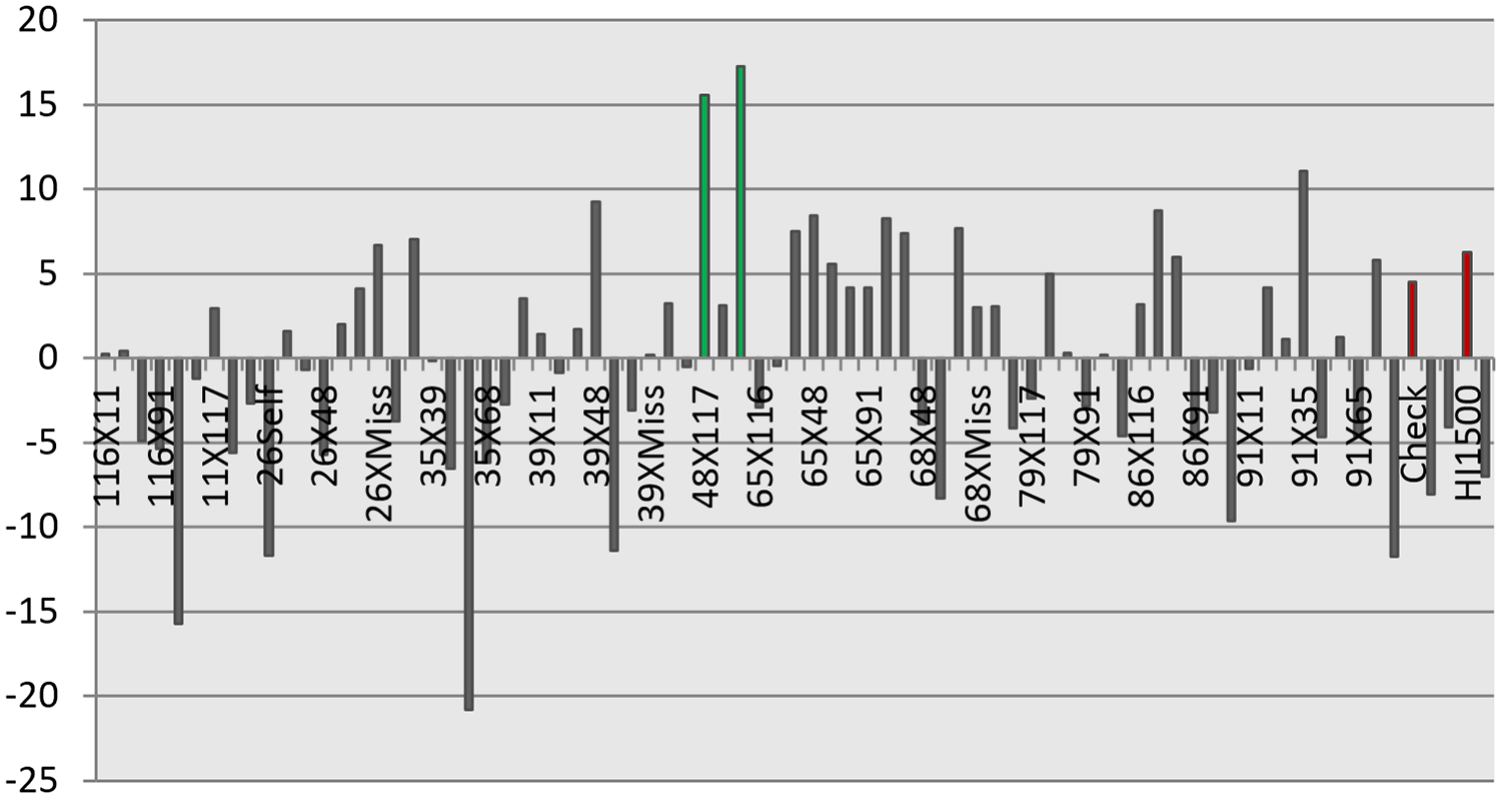
Use of best linear unbiased predictions (BLUP) for selection of lines with high yield and drought tolerance. The bars marked ‘green’ are the best performing lines 48x117 and 65 self. The bars marked ‘red’ are the check (HD 3046) and parent (HI 1500).

For the index‘TOL’, the tolerant check HD3043 occupied the first rank whereas the family hs65 was found to have top rank for MPI. Half-sib family ‘hs65’ was adjudged best for other indices also. The line with highest BLUP index was 65x65, followed by 48x117, 91x35, 39x48, 86x117 (Fig 3). Different drought indices used and the respective ranking of half-sib families, parents and checks are given in Table 5. The new lines are superior to base population and check HD3043 for grain yield while they were on par with the check HD2987. For all other traits Dunnett’s test results are given in Table 6.

**Table 5:** Dunnett’s tests comparing the performance of individual half-sib families against base population and check varieties (HD2987 and HD3043).

Against HD2987	Line	YIELD	SPAD	BIOMASS	CTVeg	CTRep	NDVI	HI
	Base	0.412	0.625	0	0.001	0.017	0.144	0
	HD3043	0.039	0.874	0.001	0.306	0.015	0.165	1
	hs116	0.872	0.36	1	0.012	0.079	0	0.149
	hs26	0.839	0.492	0.268	0.029	0.404	0.007	0.022
	hs35	0.733	0.433	0.079	0.033	0.419	0.001	0.174
	hs39	0.994	0.484	0.564	0.162	0.173	0.001	0.234
	hs48	0.998	0.767	1	0.25	0.91	0	0.004
	hs65	0.995	0.555	0.942	0.016	0.075	0	0.002
	hs68	0.999	0.835	0.86	0.014	0.641	0.001	0.004
	hs79	0.999	0.58	0.984	0.017	0.011	0	0.057
	hs86	0.889	0.526	0.968	0.133	0.217	0	0.017
	hs91	0.904	0.392	0.005	0.004	0.15	0	0.003
Against HD3043	Base	0.004	0.997	0.167	0	0.997	0.999	0
	HD2987	0.039	0.874	0.001	0.306	0.015	0.165	1
	hs116	0.005	0.972	0.001	0.389	0.948	0	0.248
	hs26	0.005	0.997	0.022	0.703	0.359	0.462	0.038
	hs35	0.004	0.991	0.081	0.753	0.345	0.055	0.286
	hs39	0.012	0.997	0.008	1	0.706	0.077	0.375
	hs48	0.014	1	0	1	0.095	0.013	0.008
	hs65	0.123	0.999	0.003	0.48	0.958	0.006	0.003
	hs68	0.016	1	0.004	0.438	0.202	0.044	0.007
	hs79	0.014	1	0.002	0.515	1	0	0.099
	hs86	0.006	0.999	0.002	0.999	0.61	0.003	0.03
	hs91	0.006	0.983	0.773	0.148	0.762	0	0.005
Against Base	HD2987	0.326	0.516	0	0.001	0.013	0.11	0
	HD3043	0.003	0.99	0.129	0	0.988	0.996	0
	hs116	0.885	1	0	0	0.599	0.002	0.002
	hs26	0.91	1	0.001	0	0.187	0.902	0.011
	hs35	0.962	1	0.002	0	0.181	0.265	0.002
	hs39	0.653	1	0	0	0.372	0.335	0.002
	hs48	0.597	0.999	0	0	0.06	0.092	0.04
	hs65	0.142	1	0	0	0.618	0.047	0.085
	hs68	0.561	0.995	0	0	0.113	0.225	0.045
	hs79	0.594	1	0	0	0.998	0.004	0.005
	hs86	0.87	1	0	0	0.315	0.029	0.013
	hs91	0.855	1	0.021	0	0.41	0.002	0.055

**Table 6:** Comparison of drought tolerance of half-sib families, base population, checks (HD 2987 and HD 3043) and parents of the original cross (HI 1500 and HUW 510) on the basis of drought indices. The indices used are TOL, GMP, MPI, MRP, REI and STI. The ranks for each index are also given. Y (RF) and Y (RI) indicate yield in grams obtained per plot from rainfed and restricted irrigation conditions.

Entries	Y(RF)	Y(RI)	TOL	GMP	MPI	MRP	REI	STI	TOL(Rank)	GMP(Rank)	MPI(Rank)	MRP(Rank)	REI(Rank)	STI(Rank)
hs116	595.7	564.9	-30.8	575.1	580.3	1.86	0.87	0.94	9	11	12	11	11	11
hs26	621	551.5	-69.5	570.1	586.3	1.88	0.88	0.96	5	12	10	10	10	10
hs35	593.9	584.3	-9.6	582.0	589.1	1.89	0.89	0.97	11	9	9	9	9	9
hs39	626	602.3	-23.8	611.6	614.1	1.97	0.97	1.06	10	8	8	8	8	8
hs48	616.5	648.8	32.3	629.7	632.6	2.03	1.03	1.12	13	3	4	4	4	4
hs65	710.8	638.8	-72.0	669.0	674.8	2.16	1.17	1.27	4	1	1	1	1	1
hs68	623	628.3	5.3	618.2	625.7	2.01	1.01	1.10	12	7	5	5	5	5
hs79	652.8	596.7	-56.1	620.3	624.8	2	1	1.09	6	6	6	6	6	6
hs86	709.3	566.4	-142.9	628.9	637.9	2.04	1.03	1.13	2	4	3	3	3	3
hs91	678.1	622	-56.1	643.0	650.1	2.08	1.09	1.18	7	2	2	2	2	2
hsib	650.9	597	-53.9	623.4	624.0	2	1	1.09	8	5	7	7	7	7
HD2987	637.5	525	-112.5	578.4	581.3	1.86	0.86	0.94	3	10	11	12	12	12
HD3043	695	462.5	-232.5	566.4	578.8	1.84	0.83	0.90	1	13	13	13	14	13
HI1500	335.7	726.1	390.4	493.7	530.9	1.73	0.63	0.68	15	14	14	15	15	14
HUW510	182	651.6	469.7	344.4	416.8	1.37	0.31	0.33	16	16	16	16	16	16
base	353.6	673.2	319.6	487.9	513.4	1.83	0.84	0.44	14	15	15	14	13	15

### Correlation for different traits in half-sib families

Significant positive correlations were observed for traits HI, biomass and NDVI with yield (Table 7). Canopy temperatures (CT) at both vegetative and reproductive stages were negatively correlated with yield. The correlation between SPAD meter reading for chlorophyll and yield was non-significant. Biomass was found to be negatively correlated with HI and NDVI. CT at both stages was found to be positively correlated with each other and a negative correlation was observed with traits like HI, NDVI and yield. However, CT and chlorophyll content was positively correlated.

**Table 7:** Correlation between different drought adaptive traits and grain yield. Traits used are biomass, Canopy Temperature (at vegetative and reproductive stages), HI,NDVI and chlorophyll content. The highlighted values indicate significant correlation at 5% level.

Biomass	1	1.000						
CT (reproductive)	2	**0.217**	1.000					
CT(vegetative)	3	**0.155**	**0.534**	1.000				
Harvest Index	4	**-0.694**	**-0.366**	**-0.262**	1.000			
NDVI	5	**-0.159**	**-0.319**	**-0.466**	**0.253**	1.000		
Chlorophyll content	6	**0.055**	**0.222**	**0.108**	**-0.033**	**-0.165**	1.000	
Yield	7	**0.179**	**-0.274**	**-0.207**	**0.514**	**0.191**	-0.01	1.000
		1	2	3	4	5	6	7

## Discussion

Recurrent selection as a population improvement strategy was first reported in maize [49–50]. The advantages of recurrent selection include rapid cycling and subsequent accumulation of favourable alleles from many parents into a single superior genotype as well as increased recombination and breaking of repulsion-phase linkages [51]. Widely popularized in maize [7, 23], recurrent selection was reported in other cereals like barley, rice, pearl millet and wheat. Under stress conditions the heritability of yield generally decline [35],although direct selection for yield is reported to be effective depending on the choice of donor [52]. Considering the variable heritability observed for grain yield across the half-sib families we used a combination of correlated secondary traits along with yield for selection under water stress.

The effect of recurrent selection on physiological traits related to drought (CT, chlorophyll content and NDVI) was discernible in nature. CT has been reported as a cheap and effective tool for predicting high wheat yield under drought stressed environments [12, 53]. Two cycles of recurrent selection resulted in cooler canopies; with a marked reduction of 1.5–4.5°C in CT at both vegetative and reproductive stages indicating enhanced tolerance to drought. Particularly CT at reproductive stage is reported to be the most important factor affecting grain yield under drought conditions [10]. The reduction in CT will affect transpiration [54] and plant water status [55] with improvement in grain filling, thus materialising high yields and drought tolerance simultaneously.

Differences in early biomass can be measured with NDVI which is a direct indication of early ground cover, a trait useful in drought adaptation [22]. The enhanced drought tolerance and yield gains achieved may also be due to better drought escape strategy by synchronising growth duration with the initial high soil moisture content [10]. The enhanced NDVI values indicating early ground cover was an indication of this. Although there was an increase in mean NDVI values after two cycles of selection, the drastic effect of drought could be read on the falling values after anthesis. The reduction in NDVI values from anthesis to maturity was comparable at RI and RF conditions. This is also an indication of reduction in biomass; one of the major yield determinant trait. The biomass reduction was evident from the narrow stem and thinner leaves observed in the half sib population.

The plant response under drought condition must be discussed in the context of *Rht* allelic profile of population. The *Rht* alleles present are *Rht-B1a* and *Rht-D1a* from HI 1500 as well as *Rht-B1a* and *Rht-D1b* from HUW 510 [56]. Apart from *Rht-D1b* (*Rht 2*), all others are gibberellin-sensitive and these genes are reported to be associated with greater expression of early vigour under drought [57]. Increased early vigour is associated with increased SLA (Specific Leaf Area) i.e thinner leaves [58] whereas SLA and SPAD reading (chlorophyll content) are negatively correlated [57]. This explains the unexpected reduction in chlorophyll content. The *Rht-D1b* allele is reported to be responsible for increase in HI and thereby higher grain yield [59]. Moreover, the wild type alleles *Rht-B1a* and *Rht-D1a* are strongly associated with QTLs for TGW (Thousand Grain Weight) and KN (Kernel Number) [60]. Although there was reduction in above ground biomass, plant height or panicle length was not affected, only the amount of straw reduced by virtue of low stem extension growth. This was beneficial because theassimilates were diverted to the growing ear thus increasing the HI. A favourable combination of gibberellin-sensitive and insensitive alleles in the population may responsible for changes in early vigour (NDVI) and associated traits (increased SLA and hence reduced chlorophyll content) as well as HI and grain yield. Increase in both early vigour and HI had a possible trade-off in reduced biomass. The reduction in biomass and chlorophyll content was an adverse effect of recurrent selection considering farmers preference of high straw varieties in the Indian sub continent.

In the present study in spite of reduced biomass content after recurrent selection, the grain yields were found to increase on an average by 17%. This gain in yield was due to changes in HI and better grain filling under stress conditions. The enhanced drought tolerance was due to better ground cover, improved transpiration efficiency and dehydration avoidance manifested through enhanced NDVI and reduced CT. In moderate warm climates, decreased CT and increased early ground cover were associated with increased yield [10, 13, 57, 61]. Under conditions of severe water stress, interplay of drought escape and tolerance mechanisms favoured by early ground cover and reduced CT along with modification of HI ensured better drought adaptation and enhanced grain filling. Moreover, for most of the drought tolerance indices the half-sib families performed better than the parents and the checks. It is interesting to note that for TOL except for two, all the other half-sib families were found to have negative values which indicate greater yield under stress conditions. The half-sib family “hs65” was found to occupy top rank in almost all the indices with very high gains in NDVI and CT.

Since the base parents (F_5_ lines) were rigidly evaluated for drought tolerance in multi-environment trials under severe and moderate stress conditions, increase in drought tolerance after intermating of lines was expected. Improving WUE is advantageous since plants can grow and yield well under water deficient conditions with more production per unit of water used [62]. In summary,after three rounds of recurrent selection, the selected half-sib progenies exhibited increased drought tolerance indicating the effectiveness of this approach. Moreover results also indicate superiority of the approach with respect to yield gain and enhanced drought tolerance over commonly practised pedigree-bulk method. The half-sib progenies with high yield and drought tolerance especially from family hs65 could be explored for the possibility of varietal development for water limited regions of North and Central India.

## Supporting Information

S1 Fig Physiological characterisation of F5 lines for canopy temperature (CT) and chlorophyll content at restricted irrigation (RI) and rainfed (RF) conditions. For brevity only selected lines, parents (158 and 159 for HI 1500 and HUW 510) and check HD 2987 given.
(DOCX)

S1 Table Environmental data of experimental locations. The maximum temperature and rainfall are given according to the growing seasons.
(DOCX)

S2 Table The best 30 F_5_ lines categorised on the basis of drought tolerance, stability across locations, yield and cultivar superiority index. Lines marked as bold are the 12 selected lines. Line 158 corresponds to resistant parent HI 1500. The ranks for each index are given at right hand side.
(DOCX)

S3 Table Pattern of crossing followed for the experiment. Each half sib family had 5 to 12 crosses, roughly confirming to half sib design. (Since some crosses are repeated, the analysis of each family was done separately.) The family hs11 was left out due to the poor seed set of cross 11X117. (Given in asterisks **.) For every cycle the same crossing pattern was followed by selecting the best lines from previous cross after phenotyping.
(DOCX)
