# Supplementary material for: Effect of Recurrent Selection on Drought Tolerance and Related Morpho-Physiological Traits in Bread Wheat
Source: PLoS One. 2016 Jun 14;11(6):e0156869. doi: 10.1371/journal.pone.0156869 (PMC4907515; doi:10.1371/journal.pone.0156869)
Supplement: S1 Fig — For brevity only selected lines, parents (158 and 159 for HI 1500 and HUW 510) and check HD 2987 given. (DOCX) [file pone.0156869.s001.docx]

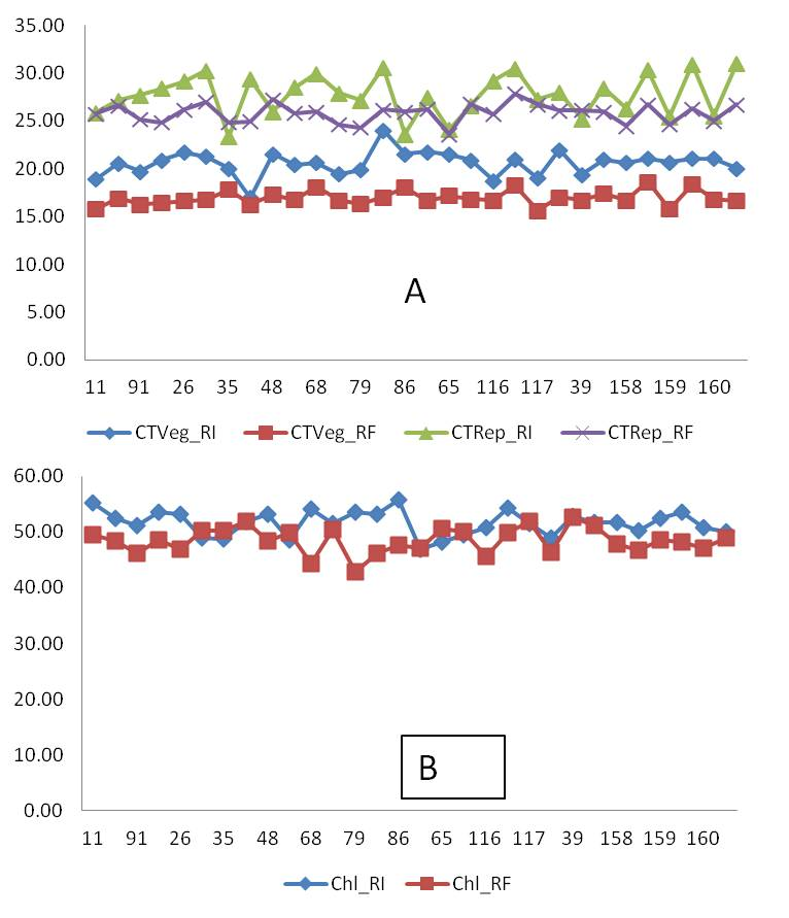


**S1Figure** Physiological characterization of F5 lines. Lines characterized for canopy temperature (CT) and chlorophyll content at restricted irrigation (RI) and rainfed (RF) conditions. For brevity only selected lines, parents (158 and 159 for HI 1500 and HUW 510) and check HD 2987 given.
