# Supplementary material for: Effect of Recurrent Selection on Drought Tolerance and Related Morpho-Physiological Traits in Bread Wheat
Source: PLoS One. 2016 Jun 14;11(6):e0156869. doi: 10.1371/journal.pone.0156869 (PMC4907515; doi:10.1371/journal.pone.0156869)
Supplement: S1 Table — The maximum temperature and rainfall are given according to the growing seasons. (DOCX) [file pone.0156869.s002.docx]

| Location | Latitude | Longitude | Altitude | T(Max) | Rainfall | Soil Type | pH | Activity done | Season |
| --- | --- | --- | --- | --- | --- | --- | --- | --- | --- |
| Delhi | 28038’N | 77009E | 228.6M | 39.50C | 42.0mm | Calcic Xe-rosol | 8.0 | Initial crossing, evaluation of advanced generations, physiological characterization, multi-environment evaluation, selection of parents, recurrent selection | Nov-April |
| Ludhiana | 30053’N | 75053’E | 241 M | 40.80C | 54.4mm | Orthic Lu-visol | 6.0 | physiological characterization, multi-environment evaluation | Nov-April |
| Powarkheda | 23009’N | 79056’E | 304 M | 410C | 35.6mm | Pellic Ve-rtisol | 7.0 | physiological characterization, multi-environment evaluation | Nov-April |
| Pune | 18031’N | 73051’E | 558 M | 400C | 57.0 mm | Chromic Vertisol | 8.0 | physiological characterization, multi-environment evaluation | Nov-April |
| Lahaul-Spiti | 32o21’N | 77o14’E | 3300M | 22.7 | 51.0mm | Sandy Loam | 7.36 | Rapid generation advancement, physiological characterization and seed multiplication | May-Oct |

**S-1Table** Environmental data of experimental locations. The maximum temperature and rainfall are given according to the growing seasons.
