# Supplementary material for: Effect of Recurrent Selection on Drought Tolerance and Related Morpho-Physiological Traits in Bread Wheat
Source: PLoS One. 2016 Jun 14;11(6):e0156869. doi: 10.1371/journal.pone.0156869 (PMC4907515; doi:10.1371/journal.pone.0156869)
Supplement: S2 Table — Lines marked as bold are the 12 selected lines. Line 158 corresponds to resistant parent HI 1500. The ranks for each index are given at right hand side. (DOCX) [file pone.0156869.s003.docx]

| TOL | MPI | MRP | REI | GMP | STI | Avg Yield | Cult-Sup | Stability | RANK |
| --- | --- | --- | --- | --- | --- | --- | --- | --- | --- |
| 143 | 11 | 11 | 11 | 11 | 11 | 11 | 11 | 11 | 1 |
| 95 | 65 | 65 | 65 | 65 | 65 | 65 | 116 | 158 | 2 |
| 135 | 158 | 116 | 116 | 116 | 116 | 116 | 65 | 65 | 3 |
| 124 | 48 | 35 | 158 | 158 | 158 | 158 | 46 | 152 | 4 |
| 88 | 117 | 39 | 35 | 35 | 35 | 35 | 39 | 46 | 5 |
| 91 | 68 | 158 | 39 | 39 | 39 | 39 | 48 | 56 | 6 |
| 51 | 116 | 26 | 26 | 26 | 26 | 26 | 158 | 90 | 7 |
| 86 | 56 | 48 | 48 | 48 | 48 | 48 | 152 | 48 | 8 |
| 141 | 91 | 86 | 68 | 68 | 68 | 68 | 79 | 18 | 9 |
| 77 | 79 | 68 | 86 | 86 | 86 | 86 | 35 | 79 | 10 |
| 66 | 46 | 91 | 91 | 91 | 91 | 91 | 68 | 68 | 11 |
| 130 | 35 | 117 | 117 | 117 | 117 | 117 | 6 | 84 | 12 |
| 8 | 39 | 79 | 79 | 79 | 79 | 79 | 117 | 149 | 13 |
| 60 | 26 | 6 | 46 | 46 | 46 | 46 | 56 | 117 | 14 |
| 57 | 152 | 46 | 56 | 6 | 56 | 56 | 26 | 112 | 15 |
| 108 | 112 | 56 | 6 | 56 | 6 | 6 | 156 | 39 | 16 |
| 106 | 86 | 134 | 134 | 134 | 134 | 110 | 110 | 91 | 17 |
| 93 | 87 | 110 | 110 | 110 | 110 | 134 | 86 | 35 | 18 |
| 4 | 6 | 64 | 64 | 64 | 64 | 64 | 149 | 69 | 19 |
| 153 | 90 | 146 | 146 | 146 | 146 | 146 | 47 | 116 | 20 |
| 157 | 126 | 126 | 126 | 126 | 126 | 126 | 91 | 156 | 21 |
| 25 | 110 | 62 | 18 | 18 | 18 | 18 | 18 | 35 | 22 |
| 39 | 13 | 18 | 62 | 62 | 62 | 62 | 126 | 47 | 23 |
| 140 | 22 | 136 | 136 | 136 | 136 | 136 | 35 | 13 | 24 |
| 35 | 18 | 159 | 159 | 159 | 159 | 159 | 69 | 121 | 25 |
| 62 | 69 | 152 | 69 | 69 | 69 | 152 | 62 | 86 | 26 |
| 26 | 134 | 69 | 152 | 152 | 152 | 69 | 84 | 87 | 27 |
| 38 | 47 | 47 | 47 | 47 | 47 | 47 | 121 | 98 | 28 |
| 85 | 133 | 20 | 149 | 149 | 149 | 13 | 101 | 12 | 29 |
| 9 | 84 | 13 | 20 | 20 | 20 | 20 | 13 | 53 | 30 |

**S-2 Table** Selection criteria of F5 lines. The best 30 F5 lines categorised on the basis of drought tolerance, stability across locations, yield and cultivar superiority index. Lines marked as bold are the 12 selected lines. Line 158 corresponds to resistant parent HI 1500. The ranks for each index are given at right hand side.
