# Supplementary material for: Effect of Recurrent Selection on Drought Tolerance and Related Morpho-Physiological Traits in Bread Wheat
Source: PLoS One. 2016 Jun 14;11(6):e0156869. doi: 10.1371/journal.pone.0156869 (PMC4907515; doi:10.1371/journal.pone.0156869)
Supplement: S3 Table — Each half sib family had 5 to 12 crosses, roughly confirming to half sib design. (Since some crosses are repeated, the analysis of each family was done separately.) The family hs11 was left out due to the poor seed set of cross 11X117. (Given in asterisks **.) For every cycle the same crossing pattern was followed by selecting the best lines from previous cross after phenotyping. (DOCX) [file pone.0156869.s004.docx]

| hs91 | hs68 | hs65 | hs39 | hs35 | hs79 | hs26 | hs86 | hs116 | hs48 | hs11 | hs117 |
| --- | --- | --- | --- | --- | --- | --- | --- | --- | --- | --- | --- |
| 91 Self | 68 Self | 65 Self | 39 Self | 35 Self | 79 Self | 26 Self | 86 Self | 116 Self | 48 Self | 11 Self | 117 Self |
| 91x117 | 68x39 | 65x91 | 39x91 | 35x79 | 79x91 | 26x79 | 86x91 | 116x91 | 48x91 | 11x117** |  |
| 91x35 | 68x26 | 65x35 | 39x26 | 35x68 | 79x86 | 26x86 | 86x39 | 116x48 | 48x117 |  |  |
| 91x68 | 68x79 | 65x26 | 39x79 | 35x65 | 79x116 | 26x116 | 86x116 | 116x117 | 48x11 |  |  |
| 91x65 | 68x116 | 65x39 | 39x116 | 35x48 | 79x48 | 26x48 | 86x48 | 116x11 | 48x65 |  |  |
| 91x39 | 68x86 | 65x79 | 39x48 | 35x39 | 79x117 | 26x117 | 86x117 | 116x26 |  |  |  |
| 91x26 | 68x48 | 65x86 | 39x117 | 35x26 | 79x11 | 26x11 | 86x11 |  |  |  |  |
| 91x86 | 68x117 | 65x116 | 39x11 | 35x11 | 79x65 |  |  |  |  |  |  |
| 91x48 | 68x11 | 65x48 | 39x35 |  |  |  |  |  |  |  |  |
| 91x116 | 68x65 | 65x117 | 39x65 |  |  |  |  |  |  |  |  |
| 91x11 | 68x35 | 65x11 |  |  |  |  |  |  |  |  |  |
| 91x79 |  |  |  |  |  |  |  |  |  |  |  |

**S3Table** Pattern of crossing followed for the experiment. Each half sib family had 5 to 12 crosses, roughly confirming to half sib design. (Since some crosses are repeated, the analysis of each family was done separately.) The family hs11 was left out due to the poor seed set of cross 11X117. (Given in asterisks **.) For every cycle the same crossing pattern was followed by selecting the best lines from previous cross after phenotyping.
